# Moving Away from Exhaustion: How Core Self-Evaluations Influence Academic Burnout

**DOI:** 10.1371/journal.pone.0087152

**Published:** 2014-01-29

**Authors:** Penghu Lian, Yunfeng Sun, Zhigang Ji, Hanzhong Li, Jiaxi Peng

**Affiliations:** 1 Department of Urology, Peking Union Medical College Hospital, Chinese Academy of Medical Science and Peking Union Medical College, Beijing, People's Republic of China; 2 Political Work Academic Department, National Defense University, Beijing, People's Republic of China; 3 Department of Psychology, Forth Military Medical University, Shaanxi, People's Republic of China; University of Leicester, United Kingdom

## Abstract

**Background:**

Academic burnout refers to students who have low interest, lack of motivation, and tiredness in studying. Studies concerning how to prevent academic burnout are rare.

**Objective:**

The present study aimed to investigate the impact of core self-evaluations on the academic burnout of university students, and mainly focused on the confirmation of the mediator role of life satisfaction.

**Methods:**

A total of 470 university students accomplished the core self-evaluation scale, Satisfaction with Life, and academic burnout scale.

**Results:**

Both core self-evaluations and life satisfaction were significantly correlated with academic burnout. Structural equation modeling indicated that life satisfaction partially mediated the relationship between core self-evaluations and academic burnout.

**Conclusions:**

Core self-evaluations significantly influence academic burnout and are partially mediated by life satisfaction.

## Introduction

Given the development and application of positive psychology in management and organization, the phenomenon of job burnout is receiving much attention. First proposed by American clinical psychologist Fredenbeger in 1974, “job burnout” describes the kind of exhaustive state, which is caused by long-time working hours, overload work, and lower satisfaction of their work, among professional workers in service careers [1]. Maslach and Jackson put forward the most widely accepted model of job burnout, which is the “Three Factors Model” that comprises emotional exhaustion, depersonalization, and reduced personal accomplishment [2].

Later, the concept of job burnout was applied to education. In 1980, Pine and Kafry found that university students experienced job burnout to a higher degree than service workers [3]. Consequently, this finding led to the concept of academic burnout in which students have no interest in studying, lack motivation, and are tired of studying [4]–[6]. The external environment and individual factors are the main causes of academic burnout [5]. The external environment factor mainly originates from excessive learning-related activities. Slivar assumed that exorbitant expectations lead to objective and subjective perceptions of continuous and immense pressure, which further results in the anxiety and weariness of the student toward studying [7]. The main individual factors that affect academic burnout are individual trait-related factors, such as self-efficacy, self-image, locus of control, self-esteem, and trait-anxiety [7]–[9]. However, studies on the factors that cause academic burnout of students, especially internal factors, are rather rare.

Core self-evaluations (CSE) have received a great attention in personality research in recent years. CSE refers to the basic evaluation of individuals of their ability and value. CSE is a higher-order factor characteristic that is above self-esteem, locus of control, neuroticism, and general self-efficacy of the four personality traits. The concept is a broader and higher level concept of personality [10]. At present, CSE research mainly focuses on organizational behavior. For example, several studies found that CSE significantly influences goal setting [11], job search behavior [12], task performance [13]–[15], interpersonal relationships [16], salary [17], and organizational commitment [18]–[19]. Studies have shown that CSE can also negatively predict job burnout [20]–[21]. Compared with studies regarding CSE and working as a variable, the relationship between CSE and learning behavior research has only emerged in the past five years. For example, a study found that the CSE has a significant negative prediction effect on test anxiety [22], and the CSE of college students can adjust the effect of intelligence on academic performance [23]. Academic burnout is an expansion of job burnout in education. Can CSE influence academic burnout? Based on existing research, self-efficacy, locus of control, and self-esteem can significantly influence academic burnout [7]–[9]. Thus, can their upper concept, CSE, predict academic burnout better? We propose:

*Hypothesis 1: CSE can predict negative academic burnout.*

If CSE can negatively influence academic burnout similar to the influence of job burnout, then what is the underlying mechanism between the two factors? Prior studies on job burnout found that all dimensions of job burnout negatively correlated with satisfaction [24]–[26]. Chinese researchers, Kang and Qu, reported that a basic linear relationship exists between the satisfaction scores of teachers and job burnout. Concretely, the degree of satisfaction increases as the degree of job burnout declines. Conversely, lower degrees of satisfaction lead to higher the job burnout [27]. Moreover, several studies have also shown that CSE can effectively predict the degree of satisfaction. Judge et al. proved CSE can directly predict the degree of satisfaction regarding living and work as well as the degree of satisfaction regarding work indirectly through operating characteristic perception, self-harmony, and achievement as objective variables [28]–[29]. In addition, Tsaousis et al. found that CSE moderately correlated with life satisfaction and adjusted to the relation between physical health and happiness [30]. Zhang et al. proved that CSE could influence organization commitment through the degree of satisfaction [19]. Thus, all these studies have consistently demonstrated that when individuals think they are more capable and valuable, they will feel more satisfied with life and work. Based on previous studies, we further propose:

*Hypothesis 2: CSE influences academic burnout by mediating life satisfaction.*

## Methods

### 2.1 Participants

The participants comprised 470 undergraduates (238 men and 232 women) from three Chinese universities. The ages of the participants ranged from 19 to 23, with a mean of 20.15 (SD = 1.76). Participants completed the questionnaires in a classroom environment, and received ¥15 as compensation. From the 470 scales that were distributed and collected, 8 unfinished scales were excluded.

All participants provided their written informed consent before completing the measures (guardians on the behalf of the minors signed the informed consent). The research described in this paper meets the ethical guidelines of the Chinese Academy of Medical Science and Peking Union Medical College. The Ethics Committee of the Chinese Academy of Medical Science and Peking Union Medical College had approved this study as well. Moreover, the research was conducted in adherence to the legal requirements of the People’s Republic of China.

### 2.2 Instruments

**2.2.1 Core self-evaluation scale (CSES).** The core self-evaluations scale (CSES), which was developed by Judge et al., is a 12-item self-report measure of core self-evaluations [29]. Items are rated from 1 (strongly disagree) to 5 (strongly agree). Examples of items include “I am confident I get the success I deserve in life” and “Sometimes when I fail, I feel worthless.” The scale scores are the sum of the ratings of the items. Relevant items were reverse-coded. In this study, the Cronbach alpha coefficient for the CSES was 0.747.

**2.2.2 Satisfaction with Life Scale.** The Satisfaction with Life Scale, which was developed by Diener and Suh, consists of five items on a seven-point rating scale (from 1  =  strongly disagree to 7  =  strongly agree) [31]. Example items include “In most ways my life is close to my ideal” and “I am satisfied with my life.” The scores are the sum of the ratings of the items. Relevant items were reverse-coded. The alpha reliability of Satisfaction with Life Scale in the present study was 0.775.

**2.2.3 Academic burnout scale.** Lian, Yang, and Wu developed the Chinese version of the Academic Burnout Scale (ABS) [32] based on the work by Schaufeli et al. [33]. Maslach and Jackson proposed the three-factor model of job burnout [2]. The ABS comprises 20 items and includes the categories of emotional exhaustion, improper behavior, and reduced personal accomplishment. Items are rated from 1 (strongly disagree) to 5 (strongly agree). Examples of items are “I feel tired when I get up in the morning” and “I have to face another day at the university” (emotional exhaustion); “I seldom range my schedule of study” (improper behavior); and “I don’t have the qualification to learn well” (reduced personal accomplishment). The ABS showed good reliability and validity. Cronbach alpha coefficients were 0.805, 0.745, and 0.780, for the three sub-scales in our study.

### 2.3 Data Analysis

**2.3.1 Structural equation modeling analysis.** To ensure that structural relations are present in the latent structured model, a two-step procedure introduced by Anderson and Gerbing was adopted to analyze the mediation effect [34]. First, the measurement model was tested to assess the extent to which the indicators of each of the three latent variables were represented. Once the confirmatory measurement model was accepted, then the maximum likelihood estimation would be used to test the structural model using the AMOS 17.0 program. The following four indices were used to evaluate the goodness of fit of the model [35]–[36]: (a) Chi square statistic (χ^2^), (b) the Standardized Root Mean Square Residual (SRMR), (c) the Root Mean Square Error of Approximation (RMSEA), and (d) the Comparative Fit Index (CFI). In this study, a model has a good fit if all the path coefficients are significant at the level of 0.05, SRMR is below 0.08, RMSEA is below 0.08, and CFI is 0.95 or more.

**Figure 1:**
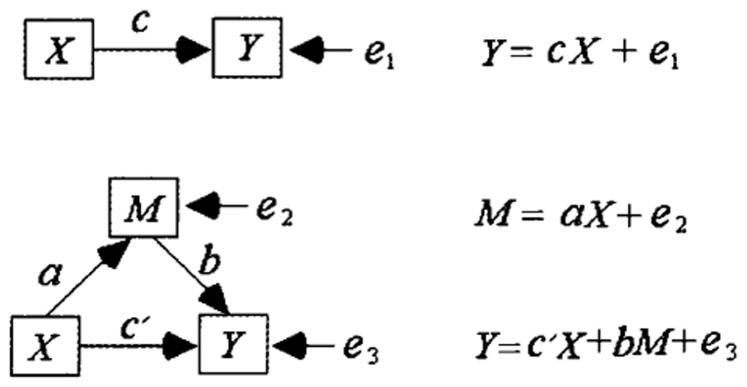
Mediation model.

**2.3.2 Mediation analysis.** We aimed to explore the trilateral relations among trait anxiety, self-frame, and decision-making. Thus, the mediation test method is emphasized. If X influences Y through variable M, then M is called the mediating variable [37]. As Figure 1 shows, assuming Y = cX+e1, M = aX+e2, and Y = c’X+bM+e3 are true, X affects Y through mediatory M, or the X-M-Y path is significant, and thus, the necessary and sufficient condition is a×b≠0 [37]–[41]. At present, three main methods can be used to verify a×b≠0. The first method is the stepwise regression [37]. This method, which is simple and easily understood, essentially requires a≠0 and b≠0. However, the following circumstance is possible. Assume coefficient a is very small (the test results are not significant), b is very large (test results is significant), and a×b≠0. Hence, if the stepwise regression method is used, one can conclude that the mediating effect is not significant. The inspective efficiency of this method is low. Moreover, the correct conclusion can easily be refused when this method is used [38]–[39]. The second method aims to test the significance of c-c’, because if c = c’+a×b, then c-c’ = a×b, then a×b≠0 is equivalent to c-c'≠0. However, to test the relationship between c-c' and 0, the standard error of c-c' must be calculated. After comparing several methods of calculating the standard error of c-c’, MacKinnon derived the formula Sc-c’ = |r_XM_|*Sc (r_XM_ is the correlation coefficient of X and M) as proposed by Clogg, Petkova, and Shihadeh [38], [40]. However, if the following condition exists: if a = 0, b≠0, a×b = 0, then Sc-c’ will be very small (a = 0, so |r_XM_| is very small), and t = c-c’/Sc-c’ can easily achieve statistical significance. Thus, in this method, incorrectly accepting the erroneous conclusion is easy. The third method tests H_0_: a×b  = 0 directly. The Sobel test is a relatively traditional method for verifying the significance of Z =  ab/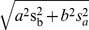 [41]. However, the Sobel test requires that ab follows a normal distribution that is always according to fact, and thus, results in reduced statistical efficacy [42]. Recently, the Bootstrap method, which uses different concepts to estimate a×b, has become popular. The Bootstrap method regards the original sample as the “entire population” by repeated sampling, and thus, generates many new sub-samples. The distribution of a×b can be acquired, and its confidence interval can be calculated. If the 95% confidence interval does not contain zero, then the null hypothesis a×b = 0 can be rejected [43]. After Taylor, MacKinnon, and Tein compared several mediating test methods with simulated data, the Bootstrap method was concluded as able to provide the most accurate confidence intervals and to have the highest statistical power [44]. Williams and MacKinnon came to the same conclusion in their similar study [39]. Therefore, this study mainly uses a structural equation and the Bootstrap method to examine the mediating effect.

## Results

Confirmatory factor analysis was used to examine whether the measurement model fit the sample data adequately. The measurement model included three latent constructs and eleven observed variables. An initial test shows the measurement model fits the data satisfactorily: χ^2^ (df  =  23, N  =  462)  =  87.90, P<0.001; RMSEA  =  0.07; and CFI  =  0.95. All the factor loadings for the indicators of the latent variables were significant (P<0.001), which indicated that the latent construct was well represented.

Furthermore, Table 1 shows the correlations of all three latent variables. CSE, life satisfaction, and academic burnout were significantly correlated with each other.

**Table 1:** Inter-correlations between core self evaluation, life satisfaction and academic burnout.

	Mean	SD	1	2
1. Core self evaluation	42.54	5.40		
2. Life satisfaction	19.91	5.67	0.417	
3. Academic burnout	52.33	10.86	–0.544	–0.510

N  =  462. All correlation coefficients are significant at p < 0.01.

In the first step, the direct effect of the predictor variable (CSE) on the dependent variable (academic burnout) without mediators was tested. The directly standardized path coefficient was significant, β  =  –0.71, P<0.001. Then, a partially mediated model (Model 1) was tested. This model contained mediators (life satisfaction) and a direct path from CSE to academic burnout. The results showed that the model did not fit the data well, χ^2^ (df = 24, N = 462)  = 120.481, P<0.001, RMSEA = 0.093, SRMR = 0.0469, and CFI = 0.936. However, an examination of the parameter estimates revealed that all the standardized path coefficients from CSE to academic burnout and life satisfaction and from life satisfaction to academic burnout were significant. Thus, according to the modification indices in Model 1, Model 2 was created through the addition of the correlations of residual terms between CSE1 and emotional exhaustion along with CSE4 and reduced personal accomplishment.

**Figure 2:**
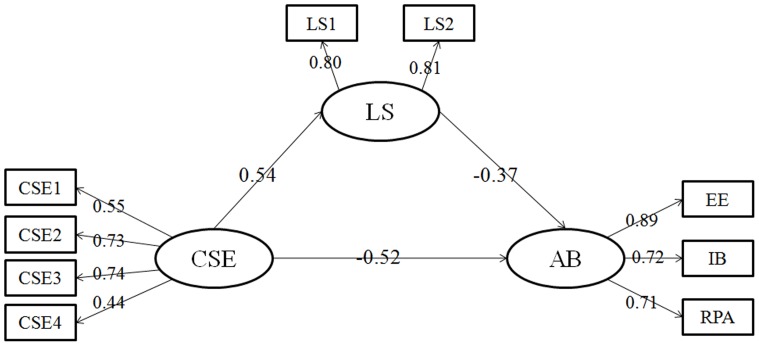
The final structural model (N  =  462). Note: Factor loadings are standardized. CSE core self-evaluations, CSE1-CSE4  =  four parcels of core self-evaluations; LS life satisfaction, LS1-LS2  =  two parcels of life satisfaction; AB academic burnout; EE emotional exhaustion; IB improper behavior; RPA reduced personal accomplishment. Form P<0.05.

After adding the correlations of the residual terms, we analyzed the final meditational model, as shown in Fig. 2. The final meditational model fit the data satisfactorily according to the following indices: χ^2^ (df = 22, N = 462)  = 50.021, P<0.001; RMSEA = 0.0319; SRMR = 0.053; and CFI = 0.981. Together, these results showed the important role of life satisfaction in the relationship between CSE and academic burnout. The effect of dispositional optimism on SWB through psychological resilience was 27.75%.

The Bootstrap estimation procedure was used to test the significance of the mediating effect of career commitment between CSE and job satisfaction in AMOS (a bootstrap sample of 1,500 was specified). Table 2 shows the indirect effects and their associated 95% confidence intervals. As shown in Table 2, CSE had significant direct impact on academic burnout. The direct effect of CSE on life satisfaction and the effect of satisfaction on academic burnout were significant. The indirect effect of CSE on academic burnout through life satisfaction was also significant.

**Table 2:** Direct and indirect effects and 95% confidence intervals for the final model.

Model pathways	Estimated	95% CI	95% CI
	effect	(Lower bonds)	(Up bonds)
**Direct effect**			
CSE→ Life satisfaction	0.543*	0.441	0.638
CSE→ Academic burnout	–0.522*	–0.402	–0.629
Life satisfaction→ Academic burnout	–0.366*	–0.245	–0.491
**Indirect effect**			
CSE→ Life satisfaction → Academic burnout	–0.199*	–0.129	–0.284

* Empirical 95% confidence interval does not overlap with zero.

## Discussion

This study used a structural equation model to construct a relation model among CSE, life satisfaction, and academic burnout. CSE was revealed to be an integrated personality variable that can directly and indirectly effect academic burnout and the mediating role of life satisfaction.

### 4.1 Impact of CSE on academic burnout

CSE provides an integrated way of thinking about issues such as how personality tendencies affect work behavior. CSE theory focuses on the more advanced structure behind several personality traits, and helps in understanding the relationship between personality tendencies and behavioral variables and for making effective predictions [11].

CSE can significantly predict academic burnout, which is consistent with previous studies [20]–[21], [45]. Hobfoll proposed the resource conservation theory, which argues that individuals attempt to acquire, preserve, and maintain their cherished resources. However, when individuals feel a disparity in terms of their input and gains, they feel threatened and are unable to adapt well, which could finally lead to burnout [46]. CSE coincides with the concept of individual resources, and is suitable for predicting burnout. CSE reflects the long-term faith of individuals on their ability to maintain a stable self and a sense of control, which are important in the evaluation of individual ability [47].

### 4.2 Mediating role of life satisfaction

Similar to the results of previous research [28]–[30], this study confirmed that CSE has a significant influence on life satisfaction. This study also proves that life satisfaction partially mediates the effects of CSE on academic burnout. CSE affects the life awareness of individuals. For example, Elliot et al. found that individuals with a positive self-view are more likely to be interested in their life than individuals who hold negative views. Their study focused on the meaning of life and the autonomy of the individual. Specifically, individuals with an optimistic self-view can look at life in a positive light as well as find pleasure and satisfaction in daily life. As a result, high life satisfaction further decreases the level of burnout [48]. Cherniss proved that job burnout is a kind of behavioral response to stress, distraction, tedious work, and job dissatisfaction. A similar study also shows that workers who are unsatisfied with their lives and jobs are more prone to burnout [24]–[26]. Moreover, college students become tired of learning because of the long-term pressure of studying. Thus, students feel unhappy and tired of their monotonous life along with their feeling of tiredness in learning. Some university students with higher CSE use more psychological resources, and thus, these students are not easily exhausted. Moreover, they have a more optimistic attitude and see the positive aspects of life quickly, which reduce the weariness of learning brought by study pressure. Thus, CSE directly and indirectly reduces the level of academic burnout.

### 4.3 Limitations

The present paper proved CSE could negatively predict academic burnout. Moreover, preliminary discussions regarding the mediating mechanism of life satisfaction between CSE and academic burnout were made. The concept of CSE was extended from employment to education. The results indicated the significance of developing the ability of students to evaluate their capability and values. Moreover, life and learning should be viewed with a positive perspective to reduce the weariness and to improve the happiness of students.

This study has some shortcomings. For instance, CSE as an integrated personality structure and personality is based on culture, and Western scholars had originally proposed CSE. Although preliminary research indicated that the CSE structure is suitable for oriental culture [19], [49], the measurement tools used in this paper were created with Western culture in mind. Therefore, a CSE tool that considers Chinese culture and characteristics must be developed. In addition, this study employed convenience sampling. All research data were derived from the several colleges in Xi'an City, which limited the results.
